# NCTC3000: a century of bacterial strain collecting leads to a rich genomic data resource

**DOI:** 10.1099/mgen.0.000976

**Published:** 2023-05-17

**Authors:** Jo Dicks, Mohammed-Abbas Fazal, Karen Oliver, Nicholas E. Grayson, Jake D. Turnbull, Evangeline Bane, Edward Burnett, Ana Deheer-Graham, Nancy Holroyd, Dorota Kaushal, Jacqueline Keane, Gemma Langridge, Jane Lomax, Hannah McGregor, Steve Picton, Michael Quail, Deepak Singh, Alan Tracey, Jonas Korlach, Julie E. Russell, Sarah Alexander, Julian Parkhill

**Affiliations:** ^1^​ Culture Collections, UK Health Security Agency, 61 Colindale Avenue, London, NW9 5EQ, UK; ^2^​ Wellcome Sanger Institute, Wellcome Genome Campus, Hinxton, Cambridgeshire, CB10 1SA, UK; ^3^​ Pacific Biosciences, 1305 O’Brien Drive, Menlo Park, CA, USA; ^†^​ Present address: Big Data Institute, Li Ka Shing Centre for Health Information and Discovery, University of Oxford, Oxford, UK, OX3 9DU, UK; ^‡^​ Present address: Quadram Institute Bioscience, Norwich Research Park, Norwich, NR4 7UQ, UK; ^§^​ Present address: Department of Veterinary Medicine, University of Cambridge, Madingley Road, Cambridge, CB3 0ES, UK

**Keywords:** National collection of type cultures, bacterial genomes, pacific bioscience long reads, genome assemblies

## Abstract

The National Collection of Type Cultures (NCTC) was founded on 1 January 1920 in order to fulfil a recognized need for a centralized repository for bacterial and fungal strains within the UK. It is among the longest-established collections of its kind anywhere in the world and today holds approximately 6000 type and reference bacterial strains – many of medical, scientific and veterinary importance – available to academic, health, food and veterinary institutions worldwide. Recently, a collaboration between NCTC, Pacific Biosciences and the Wellcome Sanger Institute established the NCTC3000 project to long-read sequence and assemble the genomes of up to 3000 NCTC strains. Here, at the beginning of the collection’s second century, we introduce the resulting NCTC3000 sequence read datasets, genome assemblies and annotations as a unique, historically and scientifically relevant resource for the benefit of the international bacterial research community.

## Significance as a BioResource to the community

Culture Collections – biological resources that maintain and supply cells or microorganisms – play a vital role in science. In addition to underpinning the scientific endeavours of a diverse, global user base through the provision of authenticated biological materials, they provide a fascinating snapshot of recent biological history. Furthermore, through active accessioning programmes, they ensure that key materials are captured for current and future generations. The genomic sequencing revolution has presented opportunities for Culture Collections to add further value to their biological materials. This both enables genomic, and potentially functional, information to guide choice of materials and provides irreplaceable historical datasets to evolutionary biologists. In a recent major project, the genome sequences of approximately half of the bacterial strains held within the National Collection of Type Cultures were determined via PacBio long-read sequencing, along with genome assemblies and annotations, and have been made publicly available. The ensuing dataset is one of the largest and highest quality genomic datasets to emerge from any Culture Collection worldwide and is already actively being used by biological community members in a diverse range of projects.

## Data Summary

The NCTC3000 project dataset was submitted to the European Nucleotide Archive under BioProject PRJEB6403. A summary of its contents at the time of writing may be seen in DataSet S1. Major components include:

1. In total, 3305 PacBio sequence read datasets from 2915 strains, representing 876 bacterial species and including 810 type strains.
2. Genome assemblies and annotations for 2238 strains.

## Introduction

The National Collection of Type Cultures (NCTC; https://www.culturecollections.org.uk/collections/nctc.aspx) [1] was established in 1920 at the Lister Institute of Preventive Medicine in Chelsea, London, in order to provide a trustworthy source of authentic bacteria for use in scientific studies. In line with these aims, the NCTC was one of the earliest collections worldwide to offer a supply service of authenticated bacteria of medical and veterinary interest to the scientific community. Since its inception, the NCTC has grown to a collection of almost 6000 strains, including type strains of 1017 species (~17 % of the collection at the time of writing) and reference strains of outstanding historical and medical interest. Since 1949, the collection has been held at Colindale in North West London, currently under the auspices of the UK Health Security Agency (UKHSA), and is under constant technical development and content augmentation for the benefit of its users.

The NCTC recently celebrated its centenary. The timing of this milestone coincided with the growing realization of the immense scientific value that historical resources such as the NCTC encapsulate. In addition to examining extant strains (key examples of which are, of course, deposited to culture collections for current and future analysis) life science researchers ever more frequently look to the recent or distant past to inform our understanding of genome composition, function and evolution. Culture collections occupy a special position in the provision of historic organisms, as custodians not only of secondary datasets derived from them but also the living organisms themselves. The global culture collection community recognize the growing need for initiatives such as the whole genome sequencing of type strains and early efforts in this area are now beginning to bear fruit.

A successful project in 2014 to short-read sequence the Murray Collection [2] (several hundred strains of mostly pre-antibiotic era *Enterobacteriaceae *, now available via the NCTC) showed how the enhancement of strain metadata with genomic characterization could add value to the strains as scientific resources. Subsequently, the Wellcome Sanger Institute and Public Health England (from October 2021, the UKHSA) developed the idea for an ambitious 5 year community resource project to long-read sequence the genomes of a further 3000 NCTC strains, with type strains a particular focus. With funding from Wellcome and support from Pacific Biosciences (PacBio), the project began in 2013, with data released into the public repositories as they were generated.

Here, we give the first report of outcomes from the NCTC3000 project. We describe the broad composition of the sequenced strains, key historical strains, and the numbers of successful sequence read, genome assembly and annotation datasets placed within the public domain to date. We describe the ongoing processes of dataset checking, validation and enhancement which will add value to the resource in the coming years. Finally, to demonstrate the utility of the sequenced strains, we touch upon some of the projects that have been made possible thus far with the public availability of the NCTC3000 datasets.

## Methods

### Sample preparation

In total, 3178 distinct NCTC strains were selected for whole genome sequencing. The strains were recovered from lyophilized ampoules, cultured on solid agar media (or alternative if required) and incubated at a temperature and atmospheric condition appropriate for the organism. Validated growth conditions on solid media are available via the NCTC online catalogues. Samples were cultured from the earliest and oldest viable lyophilized sample for each strain to avoid the occurrence of sequence mutations or loss of genetic information (e.g. plasmids) having arisen through serial *in vitro* passage. Prior to DNA extraction, the species identity of each strain was determined using MALDI-TOF.

### DNA extraction

A minimum of one DNA extraction was then performed for each strain. Due to the taxonomic breadth of the NCTC samples, encapsulating Gram-positive and Gram-negative strains, spore producers and fastidious organisms, different protocols of DNA extraction were needed to ensure recovery of high-molecular-weight genomic DNA throughout and plasmids, if present. In total, the MasterPure Complete DNA Purification Kit, the Qiagen Genomic Midi Kit with the 100/G genomic tip, and the MasterPure Gram Positive DNA Purification Kit were used as appropriate. The MasterPure Complete DNA Purification Kit was used mostly for Gram-negative bacteria, with the remaining methods used either for Gram-positives strain bacteria or those difficult to lyse and therefore providing otherwise low yields. In combination with the MasterPure Gram Positive DNA Purification Kit, DNA quantities were further boosted through the use of lytic enzymes (e.g. lysostaphin for *Staphylococcus* and mutanolysin for *Streptococcus* strains). DNA was accepted for further analysis if quantities exceeded 3 µg according to a Qubit fluorometer with a dsDNA BR Assay Kit, and electropherograms of fragment sizes showed a sharp peak of >60 kb with the Agilent 2200 TapeStation using the Genomic DNA ScreenTape, or smaller sizes of between 10 and 30 kb for strains where this size distribution was not achievable.

### Genomic sequencing

Long-read DNA sequencing was carried out at the Wellcome Sanger Institute. For each strain for which a suitable DNA extraction had been achieved, DNA was sheared to 15 kb, followed by preparation of a 10–20 kb sequencing library. Single molecule real-time (SMRT) sequencing was subsequently performed on either the PacBio RS II (prior to January 2018, hence the majority of samples) or the PacBio Sequel systems (later samples).

### Genome assembly

Genome assembly of the PacBio RS II long reads was performed using the HGAP software v3 [3] within the SMRT Analysis suite v2.3.0 [4], with fold coverage for read correction set to 30. Raw reads were then mapped back to the HGAP3 assembly, with mapping statistics generated using (the now legacy) SAMtools *bamcheck* [5]. Circularization of chromosomal and plasmid contigs within the resultant assembly was attempted with Circlator v1.1.3 [6] using HGAP3-corrected reads, followed by assembly polishing with Quiver v1 [3]. Finally, the corrected reads were mapped back to the polished assembly and mapping statistics again generated using SAMtools *bamcheck*.

An updated genome assembly pipeline was used for analysis of the PacBio Sequel-derived long reads. BAM files of uncorrected subreads were first converted to FASTQ format using SAMtools v1.6 and input to Canu v1.6 [7] to obtain a FASTA file of corrected reads. An initial HGAP4 assembly was then generated from the uncorrected subreads BAM file, with fold coverage set to 25 and an approximate genome size of 4.5 Mb. The HGAP4 assembly was then circularized with Circlator using the Canu-corrected reads. Following analysis of the (potentially) circularized assemblies with Quiver, using the uncorrected subreads, the Canu-corrected reads were mapped back to the final assembly using minimap2 v2.6 [8] and statistics generated using SAMtools. The PacBio SMRTlink modification and motif pipeline was subsequently run for each final HGAP4 assembly, using the uncorrected subreads BAM file.

### Genome annotation

For both assembly pipelines, a subsequent genome annotation was performed using Prokka v1.5 [9]. Wherever available, a genus-specific database from the RefSeq database [10] was used to guide the annotation process.

### Quality control

The species identity of each genomic dataset was assessed using two distinct methods. First, nucleotide sequences from multiple copies of the 16S and 23S rRNA genes were excised from the final assembly using profile Hidden Markov Models (*bac_16S.hmm* and *bac_23S.hmm*) available from barrnap v0.9 [11] with the *nhmmer* and *esl-sfetch* tools within the HMMER v3.2.1 software suite [12]. The two resulting FASTA files were then compared to the *16S_ ribosomal_RNA* and *LSU_prokaryote_rRNA* NCBI blast databases using the NCBI blast+ v2.11.0 *blastn* software [13].

Second, *k-mer* distributions within genome assemblies were analysed using Kraken. Each assembly was shredded into 200 bp fragments using FASTAQ (https://github.com/sanger-pathogens/Fastaq) and taxonomic classifications were assigned to fragments using Kraken2 [14] with the *MiniKraken.DB_8* Gb database as a comparator. Kraken output was parsed and collated with bespoke python3 scripts. Output of a subset of strains was also checked visually using KronaTools [15].

Genome assemblies for which at least one of these taxonomic classifications did not match the expected species (or in a small number of cases an updated species due to recent taxonomic refinements) were or will be investigated using strain-specific analysis. Priority was given to strains queried by users of the NCTC3000 dataset.

## Results

### The NCTC3000 strain set

**Fig. 1.**
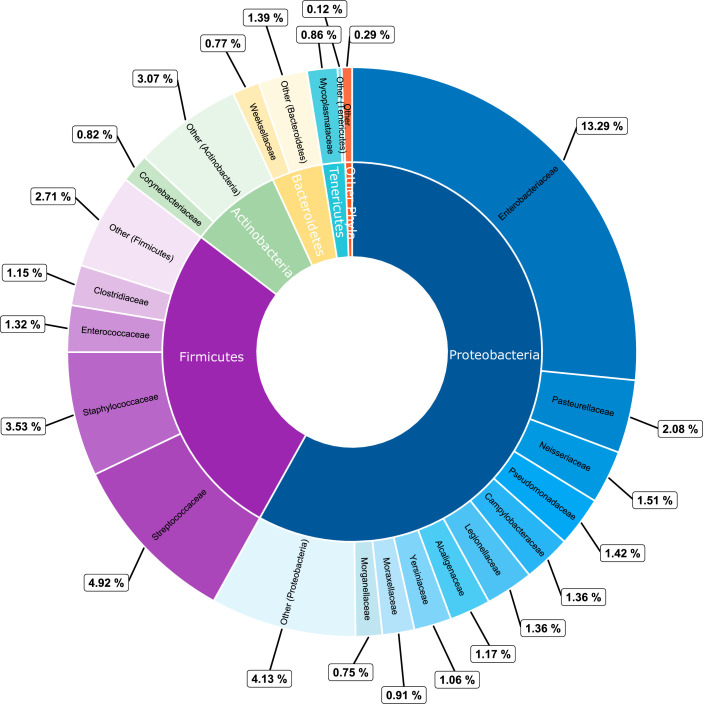
PieDonut depiction of the taxonomic distribution of the NCTC3000 dataset. The inner ring shows the five phyla (* Proteobacteria */* Pseudomonadota *, *Firmicutes */* Bacillota *, *Actinobacteria */* Actinomycetota *, *Bacteroidetes */* Bacteroidota* and *Tenericutes */* Mycoplasmatota *) with the highest frequencies from the full complement of eight phyla, with ‘Other Phyla’ representing *Fusobacteria */* Fusobacteriota *, *Spirochaetes */* Spirochaetota* and *Deinococcus-Thermus */* Deinococcota *. The plot shows, for example, that *Proteobacteria */* Pseudomonadota* (blue wedges) is the most populous phylum, comprising 58 % of the 2915 strains within the full dataset. The outer ring provides a simple breakdown of families within each phylum, showing the 17 of 96 families with a frequency >40 strains. DataSet S1 (available in the online version of this article) provides full taxonomic details of all strains, in tabular format.

Successful PacBio sequencing runs were achieved for 2915 NCTC strains, encompassing 3305 run datasets in total. Given the prior genome sequencing of NCTC 1 [16], the earliest accessioned NCTC strain within the NCTC3000 dataset was NCTC 2, a *Shigella flexneri* strain isolated in 1919, whereas the most recent accession was NCTC 13949, a *Streptococcus agalactiae* strain accessioned in 2017. The strain set includes 810 of the 1017 type strains (~80 %) accessioned to the NCTC to date. The 2915 strains were selected for their taxonomic breadth – belonging to 876 species from 219 genera, 96 families and eight bacterial phyla – while also providing depth for key species. For example, the dataset includes 280 strains of *Escherichia coli *, from NCTC 86 (isolated in ~1885) to NCTC 13919 (2016). Fig. 1 shows the frequencies of all 17 families with over 40 strains, along with the phylum designations of each family. The bias towards certain families, largely a consequence of the species composition of the collection, can be easily identified. For example, *Enterobacteriaceae* strains comprise a little over a quarter of all sequenced NCTC3000 strains.

### Data availability

All sequence reads and the majority of derived NCTC3000 project datasets were made publicly available upon generation, thereby maximizing their usability. They can be found via the ENA/GenBank/DDBJ databases by searching on BioProject PRJEB6403 or NCTC_3000, or for example by individual strain accession IDs (e.g. NCTC 2). Run datasets can be downloaded from the Sequence Read Archive (SRA), either in their uploaded HDF5 or BAM formats, or as FASTQ datasets via software such as the SRA Toolkit [17].

**Fig. 2.**
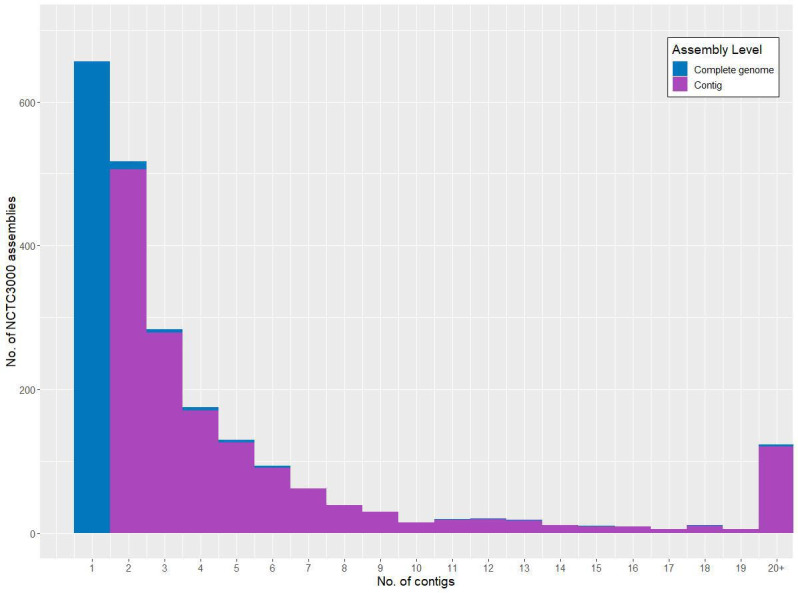
Numbers of contigs within the 2238 NCTC3000 genome assemblies publicly available prior to the time of publication. Assemblies are coloured according to their Assembly Level designation. While the 656 assemblies with a single contig comprise 95 % of the 690 ‘Complete genomes', other examples of this category can be seen with up to 29 contigs in the full dataset (NCTC 12735 *Legionella adelaidensis *, a genome with one chromosomal and 28 plasmid contigs).

Genome assemblies have been uploaded for 2238 strains to date (~77 %), with the remaining datasets set to undergo the ENA accessioning process. Of those genome assemblies accessioned prior to 12 November 2021, close to one-third (690/2238) were designated as a ‘Complete genome’, with the remainder as ‘Contigs’. The mean number of contigs per assembly was 6.95 but with a median value of 2, illustrating a left-skewed contig count distribution. This distribution can be seen in Fig. 2, such that genome assemblies with 20 or more contigs are collapsed into a single bin.

A recent analysis of the NCTC3000 Type strains estimated that prior to this study, only ~30 % of those strains had been sequenced and, where they had, many were only in draft genome form. Furthermore, approximately 12 % of cases had no prior genome sequence for any member of the species. Analysis made clear that the project is filling gaps in the bacterial genomic record.

DataSet S1 provides accession IDs for all NCTC3000 datasets uploaded to date. However, users should note that the dataset is constantly under review, as outlined in the Data validation section below, and this document should therefore be considered as a potentially dynamic record.

### Dataset validation

The Quality Control procedures outlined within the Methods section, along with welcomed feedback from users, are being used to refine the dataset. While the majority of validation analyses provide both rRNA markers and *k-mer* profiles consistent with the expected species of the strain in question, samples have been identified where one or more do not match the expected species or they identify more than a single species. For example, analysis of the genome assembly for *Staphylococcus aureus* strain NCTC 13131 showed eight rRNA loci, six with high sequence similarity to known *Staphylococcus aureus* sequences and the remaining two to a *Microbacterium *, suggesting contamination of the sequenced sample with a *Microbacterium* strain. Further analysis of the genome assembly showed that seven of its 18 contigs could be attributed to *Staphylococcus aureus *. In conclusion, a revised genome assembly (accession ID GCA_900458265.2) was submitted to the ENA.

In addition to approximately 14 cases of sample contamination identified to date, two cases were identified as strain swaps. In each case, two strains contemporaneously prepared for sequencing within a small strain batch were inadvertently labelled as one another. Fortunately, genomic analysis in tandem with sequencing manifests can be used to identify such eventualities. In other cases, an identified single species may be a close relative of the expected species. Such cases can indicate that a taxonomic refinement to the species of the original NCTC strain, which often will have been specified on morphological or biochemical data rather than DNA-based methods, may be necessary. Other cases may be complicated by suspected erroneous or ambiguous datasets within, for example, the rRNA databases, requiring additional analysis to resolve the identification. Due to the size of the NCTC3000 dataset, this process is ongoing, with completion expected within 2023.

### The NCTC3000 dataset encompasses key strains of historical interest

**Fig. 3.**
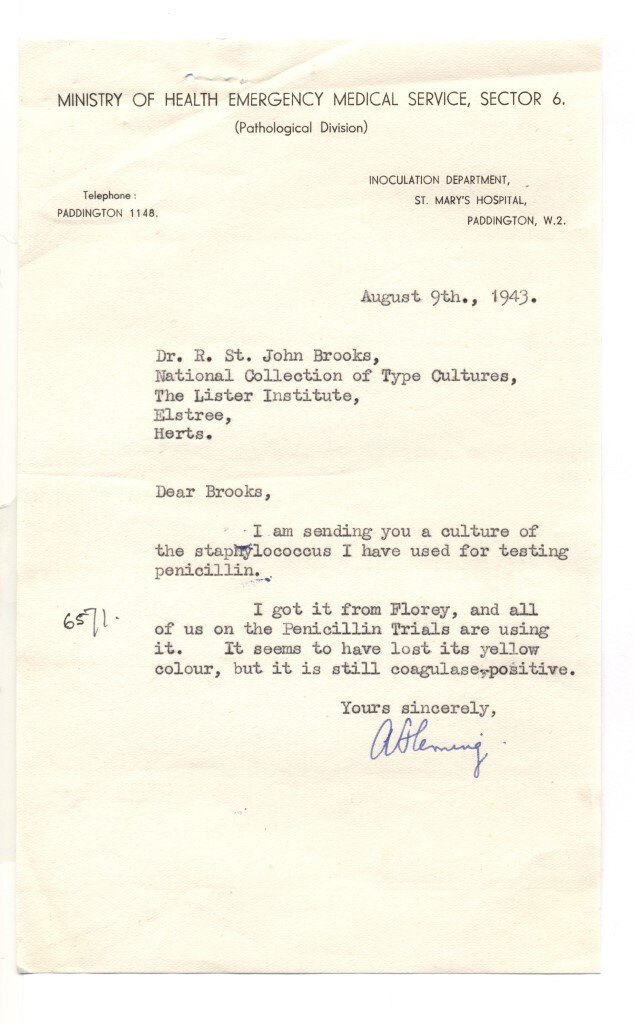
Letter to the National Collection of Type Cultures accompanying the accession NCTC 6571, the ‘Oxford Staphylococcus’ associated with the discovery of penicillin as a therapeutic agent. The genome assembly of this strain can be found within the NCTC3000 dataset, under accession GCA_900457695.1.

Inclusion of a strain within the NCTC is frequently a commentary on its utility or historical relevance. Indeed among the bacterial strains sequenced by the NCTC3000 project are many that represent significant milestones and discoveries of microbiology and bacteriology. For example, NCTC 86 is the *Escherichia coli* strain originally isolated in 1885 as ‘Bacterium coli commune’ by Theodor Escherich. The product of many other eminent scientists’ work is also captured herein; NCTC 2665 *Micrococcus luteus* and NCTC 6571 *Staphylococcus aureus* were both deposited by Sir Alexander Fleming as a consequence of his discovery of lysozyme in 1922 and research during the penicillin trials at Oxford during the 1940s, respectively. Fig. 3 shows an image of the letter accompanying the accession of what was to become NCTC 6571, an example of the historical metadata associated with the strains, and now their genomic datasets.

Other newly sequenced strains reflect remarkable events at the interface of infectious disease and human history. Following the successful sequencing and description of NCTC 1 *Shigella flexneri* [16] and NCTC 30 *Vibrio cholerae* [18], both isolated from British soldiers who fought in World War One, the NCTC3000 dataset includes genomic datasets from additional strains isolated during times of conflict. For example, NCTC 160 *Salmonella enterica* subsp *. enterica* serotype Typhi was isolated by Sir Almroth Wright from an outbreak of typhoid during the Second Boer War, South Africa in 1900–1901. Several *Clostridium* spp. (e.g. NCTC 275 *Clostridium sporogenes *) were isolated from anaerobic infections of war wounds gained in World War One by the under-recognized some-time bacteriologist Muriel Robertson. Many of these strains poignantly bear the names of the soldiers from whom they were isolated.

The NCTC3000 dataset also encompasses historically important outbreak strains such as NCTC 11192 *Legionella pneumophila *, isolated in 1976 during the first Legionella outbreak in individuals returning from a convention of the American Legion in Philadelphia, USA, with a pneumonia-like illness. The dataset also captures strains key to the ever-growing threat of antimicrobial resistance, including the first known methicillin-resistant *Staphylococcus aureus* (MRSA) and vancomycin-resistant enterococci (VRE) strains to be isolated in the UK (NCTC 10442 *Staphylococcus aureus* deposited by Dr Margaret Patricia Jevons, and NCTC 12201, NCTC 12202 and NCTC 12204 by Dr Anne H. C. Uttley, respectively).

Several sets of strains were isolated throughout the twentieth century, such as the typing strain panels NCTC 8180 – NCTC 8188 *Streptococcus agalactiae* (eight successfully sequenced strains, including the type strain) and NCTC 11886 – NCTC 11911 *Streptococcus pneumoniae* (18 strains). While these sets do not offer a comprehensive catalogue of contemporaneous circulating strains, each offers a valuable snapshot of the evolution of bacterial pathogens of medical importance, of which the 26 new genomic datasets can now offer a more detailed view.

While predominantly a bacterial collection of medical importance, several newly sequenced strains are type strains of especially multifarious provenance, from ground water samples at Mount St. Helens 1 year following its eruption in 1980 (NCTC 11990 *Legionella spiritensis* and NCTC 11988 *Legionella sainthelensi *) to isolates such as NCTC 7523 *Kocuria rosea *, obtained from the Kral Collection (1890–1911; Prague, The Bohemian Kingdom, modern day Czech Republic), one of the world’s first microbial collections.

In terms of historical veterinary importance, the second arm of the NCTC’s custodial remit, this definition is perhaps most stretched in consideration of NCTC 4736 *Kocuria viridans* and NCTC 6197 *Proteus mirabilis *, the latter of which was sequenced successfully within the NCTC3000 project. These strains were both isolated from the pet pedigree Chow Chow (‘Rex’) belonging to Ralph T. St John-Brooks, the first NCTC curator, at various points in its life. Unfortunately, the purpose of collecting these isolates is unknown.

Each strain held by the NCTC carries with it its own history. Whether any given strain is a fragment of the legacy of human conflict, a reflection of one man’s relationship with his dog, or a key event in the arms race between humanity and its numerous bacterial threats, it is to be hoped that by performing high-quality sequencing of these strains to accompany the biological agents themselves, plus their associated metadata, the history and legacy of each strain is made more valuable to those researchers who will use it. Table 1 shows NCTC3000 genome assembly information for many of the strains discussed above, which illustrate the century of NCTC strain collecting undertaken to date.

**Table 1.** Examples of NCTC strains with key historical and scientific value

Accession ID	Description	Genome assembly	No. of contigs
NCTC 86	* Escherichia coli* strain isolated in 1885 as ‘Bacterium coli commune’ by Theodor Escherich	GCA_900699165.1	1
NCTC 160	* Salmonella enterica* subsp. *enterica* serotype Typhi strain isolated by Sir Almroth Wright from an outbreak of typhoid during the Second Boer War	Pending accession	5
NCTC 275	* Clostridium sporogenes* strain from a World War One soldier’s case of gas gangrene at Flanders in 1914, deposited by Muriel Robertson	GCA_900447115.1	2
NCTC 2665	* Micrococcus luteus* strain deposited by Sir Alexander Fleming following his discovery of lysozyme in 1922	GCA_900475555.1	1
NCTC 6197	* Proteus mirabilis* strain isolated from the pet pedigree Chow Chow (‘Rex’) belonging to Dr Ralph T. St John-Brooks, the first NCTC curator	GCA_900454995.1	5
NCTC 6571	* Staphylococcus aureu* s strain (the ‘Oxford Staphylococcus’) used in penicillin assays, deposited in 1943 by Sir Alexander Fleming	GCA_900457695.1	2
NCTC 7523	Type strain of *Kocuria rosea *, obtained from the Kral Collection, one of the world’s first microbial collections	Pending accession	1
NCTC 10442	First methicillin-resistant *Staphylococcus aureus* (MRSA) strain deposited in 1966 by Dr Margaret Patricia Jevons	GCA_900458485.1	3
NCTC 11192	* Legionella pneumophila* Philadelphia 1 serotype 1 isolate from the first Legionella outbreak in 1976	GCA_900452735.1	2
NCTC 11988, NCTC 11990	Type strains of *Legionella sainthelensi* and *Legionella spiritensis *, respectively, isolated from ground water samples at Mount St. Helens 1 year following its eruption in 1980	GCA_900637685.1, GCA_900186965.1	1, 1
NCTC 12201, NCTC 12202, NCTC 12204	First vancomycin-resistant enterococci (VRE) strains to be isolated in the UK by Dr Anne H. C. Uttley	GCA_900447835.1, GCA_900448035.1, GCA_901482495.1	6, 5, 3

### NCTC3000 datasets underpin scientific endeavour

**Fig. 4.**
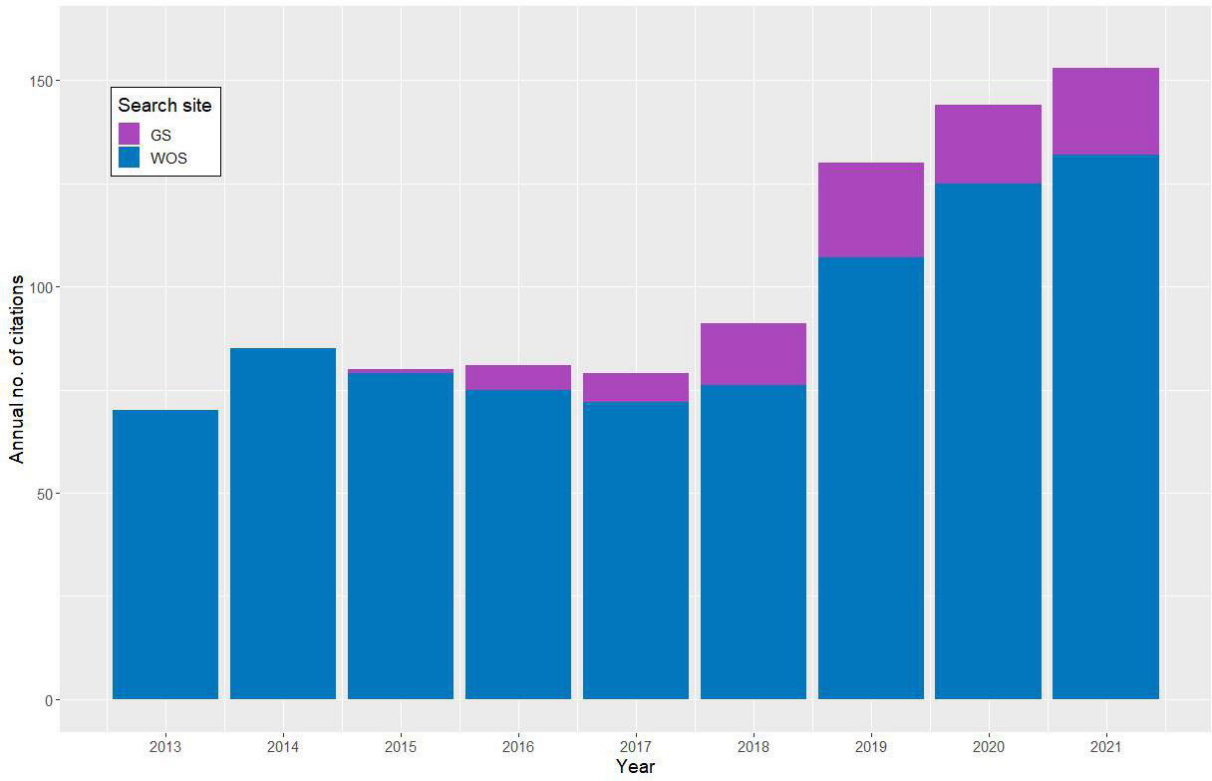
Numbers of citations of ‘NCTC’ in Web of Science (WOS; blue bars), and ‘NCTC3000’, ‘NCTC 3000’, ‘NCTC_3000’ or ‘PRJEB6403’ in Google Scholar (GS; violet bars) between the years 2013 and 2021.

There is growing evidence that the genomic datasets generated within the NCTC3000 project have already begun to make an impact on the bacterial research landscape, enhancing scientific projects and adding value to the strain collection. Searching the Google Scholar resource on 4 August 2022 indicated over 2000 hits per year for the term ‘NCTC’, with a total of 18 900 records in the period 2013–2021. Limiting the search to Web of Science Core Collection journals in the same period shows a more modest sum of 821 articles. This smaller frequency is due to Web of Science searching journal article fields such as Title, Abstract and Keywords, whereas Google Scholar searches the entire text of a wider range of document types. However, both sources hint at an increase in the annual number of NCTC-related articles published since the NCTC3000 genomic datasets have been publicly available (see Fig. 4 for Web of Science statistics).

Additional searching of the NCTC3000-related terms (‘NCTC3000’, ‘NCTC 3000’, ‘NCTC_3000’ and ‘PRJEB6403’) in Google Scholar shows that 92 articles can be definitively linked to the NCTC3000 project between 2015 and 2021. A cursory examination of Google Scholar and Web of Science ‘NCTC’ hits suggests that some articles that used NCTC3000 genomic datasets did not specifically reference this term, instead using the more general ‘NCTC’, probably due to the lack of a prior NCTC3000 publication to cite.

Fig. 4 shows the results of the Web of Science ‘NCTC’ and Google Scholar NCTC3000-related hits from 2013 to 2021, spanning all full years since the NCTC3000 project began to date. Consequently, there is clear evidence that the biological research community is making use of the data. The titles of article hits suggest that the uses are varied, encompassing analysis of individual strains, species or population analyses and software tool development, which we will illustrate in the next section.

### NCTC3000 datasets have many-faceted uses

The composition of the NCTC3000 sequenced strains, exhibiting both taxonomic breadth and, in some cases, species depth, lends the dataset well to a range of use cases. To date, these uses predominantly fall into three categories. First, the use of a single strain dataset, particularly one of historical significance, can provide an invaluable backdrop to a more modern strain set. Second, a group of strains from a common species can, either on their own or in combination with additional genome sequences, provide detailed information on the functional capability, or evolution, of the species. Finally, the taxonomic range of the dataset can be exploited in the development, testing or demonstration of software tools that aim to enhance the genome sequences by utilizing information within the genome assemblies, the sequence read datasets or the two combined. Here, we briefly describe three projects, which together provide one instance of each of these three use cases, illustrating the versatility of the NCTC3000 dataset.

### Population genomic analysis using an historical, single strain dataset

The *Klebsiella pneumoniae* strain NCTC 9494 was accessioned into the NCTC in 1954, obtained from the US Communicable Disease Center (CDC; since renamed Centers for Disease Control). Lam *et al.* [19] investigated the evolutionary history of a set of hypervirulent clonal-group CG23 *Klebsiella pneumoniae* strains, a lineage associated with severe liver abscess infections. The NCTC3000 dataset for NCTC 9494 served as the earliest of a small set of genome references for the sublineage CG23-I most closely associated with the clinical focus of the study. Through a series of detailed computational analyses of 98 human and equine-associated strains, the authors presented an improved evolutionary history for CG23 and new information on the prevalence of key virulence loci such as integrative conjugative elements (ICEs). In particular, the ICEKp10-encoded colibactin synthesis locus (*cbl*), absent in NCTC 9494, was found to precede the emergence of CG23-I in ~1928, and is hypothesized to have supported the success of this sublineage.

### Gene family investigation of a multi-strain species dataset

The type VII secretion system (T7SS) found in many Gram-positive bacteria is believed to play a key role in inter-bacterial competition in *Firmicutes *. Important T7SS components include co-localized arrays of closely related but non-identical immunity genes that provide protection against the toxic polymorphic effector protein, EsaD. A recent study aimed to investigate the evolution of these gene families [20]. Using 31 *Staphylococcus aureus* genome assemblies from the NCTC3000 dataset, where the strains were all predicted to derive from Clonal Complex 8, a range of computational analyses was used to uncover strong evidence for extensive homologous recombination at the *ess*/*T7SS* locus. In particular, two expansion and five loss events were inferred within the NCTC strains, leading to three mosaic forms of the *esaG* gene family. Analysis of additional *Streptococcus mitis* genomes presented evidence for a similar phenomenon in the T7SS *tipC* gene family (though not definitively in the *S. aureus tsaI* gene family), raising the possibility that homologous recombination plays an important role in enabling the rapid remodelling of the T7SS locus, including the acquisition of novel immunity genes.

### Software tool development using multi-species datasets

Developing new computational approaches and software tools for the analysis of biological datasets can be aided significantly by the availability of high-quality real datasets for benchmarking and testing. Given its taxonomic breadth and foundation on long-read sequence reads, the NCTC3000 dataset is well placed to fulfil such a role. One area of frequent recent use has been in long-read genome assembly and refinement. For example, the genome assembly tool Raven used NCTC3000 genome assemblies to determine empirical constants used within its workflow [21] whereas B-assembler, a bacterial genome assembler, used NCTC3000 read datasets and assemblies for 14 strains spanning eight genera to evaluate and compare its results [22]. Moreover, the mosaicFlye tool, aimed at resolving long mosaic repeats for genome assembly improvement, used 20 NCTC3000 read datasets to demonstrate advances in this area [23] and Asgan used 21 NCTC3000 read datasets to demonstrate levels of concordance between genome assembly synteny paths found within the results of using different assembly tools [24].

## Discussion

The NCTC is one of the world’s oldest bacterial collections. The NCTC3000 project was conceived as a mechanism to add value to its strains through genome sequencing and analysis. Use of the NCTC3000 dataset within the biological community is growing, as evidenced by citations data, confirming the benefit of genome sequencing of historical collections.

Currently, all NCTC strain numbers are included within NCTC3000 ENA/GenBank/DDBJ records. Therefore, useful or novel features discovered through genomic analyses of the dataset may be linked back to their biological source. A key focus for NCTC over the coming years will be to fully integrate the NCTC3000 dataset with the strains themselves, thereby enabling bi-directional links. A series of species-focused projects has already led to a wealth of detailed annotation data and a major recent initiative has led to the digitization of NCTC historical strain metadata. One ambition is therefore to develop a database that combines genetic and putative functional information with links to the ENA/GenBank/DDBJ datasets and to the strain metadata such that these may be dynamically served to users via the NCTC website. A major consequence of this would be to give users the highest level of discriminatory power when choosing a strain or genomic dataset for their own projects, for example the ability to choose a strain from a particular lineage, geographical region or accession time, and possessing a required plasmid or gene. Such questions are already posed to NCTC staff from time to time; the ability to use computational methods to deliver these data routinely is highly attractive.

## Supplementary Data

Supplementary material 1
